# Breast Ultrasound Image Segmentation Integrating Mamba-CNN and Feature Interaction

**DOI:** 10.3390/s26010105

**Published:** 2025-12-23

**Authors:** Guoliang Yang, Yuyu Zhang, Hao Yang

**Affiliations:** School of Electrical Engineering and Automation, Jiangxi University of Science and Technology, Ganzhou 341000, China; ygliang30@126.com (G.Y.);

**Keywords:** breast ultrasound images segmentation, mamba, hybrid attention enhancement mechanism, cross fusion module

## Abstract

The large scale and shape variation in breast lesions make their segmentation extremely challenging. A breast ultrasound image segmentation model integrating Mamba-CNN and feature interaction is proposed for breast ultrasound images with a large amount of speckle noise and multiple artifacts. The model first uses the visual state space model (VSS) as an encoder for feature extraction to better capture its long-range dependencies. Second, a hybrid attention enhancement mechanism (HAEM) is designed at the bottleneck between the encoder and the decoder to provide fine-grained control of the feature map in both the channel and spatial dimensions, so that the network captures key features and regions more comprehensively. The decoder uses transposed convolution to upsample the feature map, gradually increasing the resolution and recovering its spatial information. Finally, the cross-fusion module (CFM) is constructed to simultaneously focus on the spatial information of the shallow feature map as well as the deep semantic information, which effectively reduces the interference of noise and artifacts. Experiments are carried out on BUSI and UDIAT datasets, and the Dice similarity coefficient and HD_95_ indexes reach 76.04% and 20.28 mm, respectively, which show that the algorithm can effectively solve the problems of noise and artifacts in ultrasound image segmentation, and the segmentation performance is improved compared with the existing algorithms.

## 1. Introduction

Breast cancer is currently one of the most common malignant tumors in women and has become a major killer of women’s health [1]. The latest data indicate that breast cancer has become the leading cause of cancer incidence among women [2]. Therefore, early diagnosis and treatment of breast cancer are crucial for patients. Ultrasound imaging technology has become one of the most common methods for early diagnosis of breast cancer due to its affordability, ease of operation, and noninvasiveness [3]. BUS (Breast Ultrasound Scan) images can be analyzed to well diagnose whether the breast is diseased and the extent of the lesion, and to better prevent the disease. However, manual segmentation is often time-consuming, laborious, and subjective, and breast ultrasound images have many inherent defects such as speckle noise, artifacts, low contrast, and low resolution, etc., all of which make the segmentation of breast ultrasound images very challenging. Therefore, the use of computer-aided diagnosis, through the automatic segmentation of breast ultrasound images, can accurately identify the breast ultrasound image of the skin, glands, and tumors and other tissue areas, to provide doctors with more accurate information about the lesions, so as to assist the doctor in carrying out more accurate diagnosis and treatment [4].

Breast ultrasound image segmentation belongs to an important branch of medical image segmentation, and its main methods can be categorized into those based on traditional image processing and those based on deep learning. The traditional image processing methods can be mainly categorized into region growing algorithms [5] and the threshold segmentation method [6]. However, methods based on traditional image processing are only suitable for simple image segmentation with high imaging quality, but the segmentation effect is poor for complex environments with high noise and many artifacts. Additionally, with the development of deep learning, various deep learning-based breast ultrasound image segmentation methods have been enthusiastic.

Convolutional Neural Networks (CNN) are an end-to-end deep learning method that can extract deep features well and have made amazing progress in breast ultrasound image segmentation [7]. Among the convolutional neural network models, Unet [8] proposed by Ronneberger et al. is the most classic. Unet is a deep learning network designed for medical image segmentation with a symmetric U-shaped structure as well as jump connections. Later on, a large number of CNN-based models emerged, such as U-net++ [9], FPN [10] and DeepLabv3+ [11]. Based on these approaches, Shareef et al. [12] used deterministic kernels to adapt breast segmentation structures and designed dual encoders to fuse information from different scales to segment small breast tumors. Yap et al. [13] investigated three CNN-based approaches for breast ultrasound lesion detection. Hu et al. [14] proposed to combine dilated convolutional networks with phase-based active contouring models to automatically segment breast lesion regions. Zhu et al. [15] developed a second-order subregion network for breast lesion segmentation by utilizing the second-order statistics of multiple feature subregions. All these CNN-based methods have some limitations, and the local operation of their convolution can make the network under- or over-segmented when performing breast ultrasound image segmentation.

Transformer [16] has a global dependency modeling capability to capture long-range dependencies through a self-attention mechanism, and its superior performance has achieved excellent performance in medical image segmentation. Chen et al. [17] combined the advantages of Transformer and Unet and used Transformer architecture as an encoder, which well improved the medical image segmentation task. Mo et al. [18] proposed Hover-trans network for breast tumor diagnosis in breast ultrasound images using Transformer. Cao et al. [19] proposed a pure Transformer network model with Unet architecture, which uses a hierarchical self-attention mechanism with a shift window, namely Swin Transformer, as encoder and decoder. However, Transformer has a better performance; it requires pre-training on many datasets, and its high computational complexity leads to a large overhead for training and inference.

Recent developments in the state space model (SSM), especially structured SSM (S4) [20] provide a very promising solution for dealing with long sequences. The Mamba model enhances the S4 model with selective mechanisms and hardware optimizations, which present excellent performance in dense data domains [21]. The introduction of the Cross-Scan Module (CSM) in Visual Mamba further enhances the applicability of Mamba to computer vision tasks by traversing the spatial domain and converting the visual images into an ordered sequence of patches [22]. Wang et al. [23] proposed the MambaUnet, which can synergize the capabilities of Unet and Mamba in medical image segmentation to improve the segmentation performance.

Inspired by the above, a breast ultrasound image segmentation model integrating Mamba-CNN and feature interaction is proposed to address the problems of speckle noise and many artifacts in breast ultrasound images. The main contributions of this paper are as follows: (1) a novel network is designed to capture long-range dependencies with linear time complexity using the Mamba module, and the feature maps are upsampled step by step using transposed convolution [24], which recovers the spatial information of the images while improving their resolution; (2) a hybrid attention enhancement mechanism, HAEM, is designed as the bottleneck module of the model, which is used for feature maps by spatial attention and channel attention for feature selection of the feature map, focusing on more critical regions and features for better information extraction; (3) A cross-fusion module, CFM, is constructed to interact the output of the encoder as well as the output of the decoder, which organically combines the semantic information of the shallow feature map as well as the spatial information of the deeper feature map to improve the segmentation capability effectively; (4) Compared with several mainstream medical segmentation models in the BUSI and UDIAT datasets shows better segmentation performance, which provides some application value for early diagnosis of breast tumors.

## 2. Materials and Methods

With the proposal of the state space model Mamba, more visual Mamba models have been used in the field of image processing. The Mamba model alleviates the modeling constraints of convolutional neural networks through global sensing field and dynamic weighting, and avoids the secondary computational complexity of Transformer while possessing the long sequence modeling capability of Transformer. The Mamba model effectively combines with CNN, which is very helpful to deal with the problems of blurred boundaries and severe noise in breast ultrasound image segmentation.

### 2.1. General Structure of the Network Model

**Figure 1:**
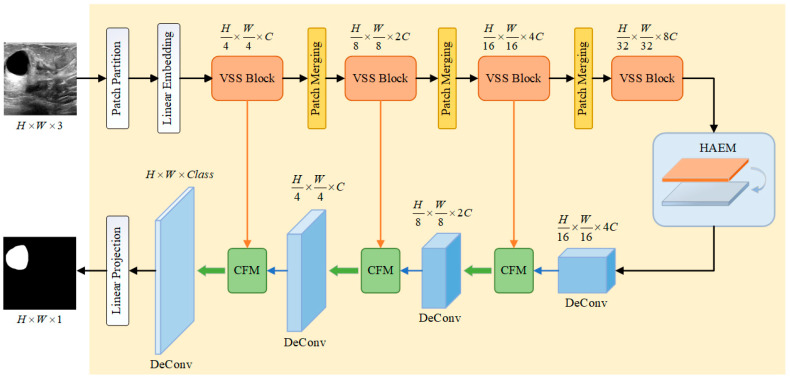
Breast ultrasound image segmentation model integrating Mamba-CNN and feature interaction.

To solve the problems of much speckle noise, serious artifacts, and blurred boundaries in breast ultrasound image segmentation, this paper proposes a breast ultrasound image segmentation algorithm that incorporates Mamba-CNN and feature interactions, and the overall structure of its network is shown in Figure 1.

The main structure of the model is divided into four parts, i.e., encoder (Visual State Space model, VSS), HAEM, Transpose Convolution (Deconvolution) and CFM.

The input image of size H×W×3 is first grayscaled and decomposed into ViT-like patch blocks [25], which are then integrated into a one-dimensional sequence of size $\frac{H}{4}\times \frac{W}{4}\times 16$. Afterwards, the dimensions are scaled to an arbitrary size that can be represented by C by means of a linear embedding layer, i.e., $\frac{H}{4}\times \frac{W}{4}\times C$. These one-dimensional sequences are then processed through multiple VSS blocks and a patch merging layer [26] to create layered features. The VSS blocks focus on feature extraction and learning, while the Patch Merging layer downsamples the features and increases the channel dimensions. The output dimensions of each layer of the encoder are $\frac{H}{4}\times \frac{W}{4}\times C$, $\frac{H}{8}\times \frac{W}{8}\times 2C$, $\frac{H}{16}\times \frac{W}{16}\times 4C$, and $\frac{H}{32}\times \frac{W}{32}\times 8C$, respectively. The HAEM does not change the output dimension of the last layer of the encoder. The decoder section uses transposed convolution to upsample the feature map, which can maintain the same dimension as the encoder output, and undergoes CFM for feature interaction. Where the encoder part, i.e., VSS module, loads VMamba Tiny pre-trained weights.

### 2.2. Encoder Module VSS

The VSS block originated from the State Space Model (SSM). Specifically, in SSM, each channel of the input vector x is mapped to the output vector y and then transformed through a high-dimensional latent state h. This process is jointly achieved by the projection process and the selection mechanism. This model can be described as:

(1) $$h^{\prime }\left(t\right)=Ah\left(t\right)+Bx\left(t\right)$$

(2) $$y\left(t\right)=Ch\left(t\right)+Dx\left(t\right)$$

where $x\left(t\right)\in \mathbb{R}$, $y\left(t\right)\in \mathbb{R}$, $h\left(t\right)\in {\mathbb{R}}_{\;⠀}^{N}$, $A\in {\mathbb{C}}_{\;⠀}^{N\times N}$, $B\in {\mathbb{C}}_{\;⠀}^{N}$, $C\in {\mathbb{C}}_{\;⠀}^{N}$, $D\in {\mathbb{C}}_{\;⠀}^{1}$. A, B, C, and D are all weighting factors.

To integrate SSM into a deep learning model, the continuous time model SSM must be discretized, and given the time scale parameter $\Delta \in {\mathbb{R}}_{\;⠀}^{D}$, the discrete model of SSM can be obtained by transforming it with a zero-order holder:

(3) $$h\left(t\right)=\bar{A}h_{k-2}^{\;⠀}+\bar{B}x_{k}^{\;⠀}$$

(4) $$y_{t}^{\;⠀}=Ch_{k}^{\;⠀}+\bar{D}x_{k}^{\;⠀}$$

(5) $$\bar{A}=e_{\;⠀}^{\Delta A}$$

(6) $$\bar{B}=\left(e_{\;⠀}^{\Delta A}-I\right)A_{\;⠀}^{-1}B$$

(7) $$\bar{C}=C$$

where $B\in {\mathbb{R}}_{\;⠀}^{D\times N}$, $C\in {\mathbb{R}}_{\;⠀}^{D\times N}$. Approximating $\bar{B}$ using a first-order Taylor series, i.e., $\bar{B}=\left(e_{\;⠀}^{\Delta A}-I\right)A_{\;⠀}^{-1}B\approx \left(\Delta A\right){\left(\Delta A\right)}_{\;⠀}^{-1}\Delta B=\Delta B$.

**Figure 2:**
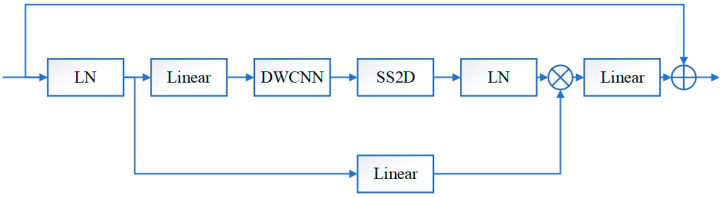
Visual state space model VSS.

The CSM is further introduced in the visual Mamba model, and then the convolution operations are integrated into the VSS block. The structure of the visual state space module VSS is shown in Figure 2, where LN denotes layer normalization, Linear denotes a linear layer, DWCNN denotes depth convolution, and SS2D is 2D selective scanning. In the VSS block, the input features are first layer normalized to the linear embedding layer and then forked into two branches into different layers. One of the branches undergoes deep convolution [27] and SiLU [28] activation, enters the SS2D block, undergoes layer normalization again, merges with the other branch after SiLU activation, and finally enters the linear layer as well as residual concatenation to obtain the output. Unlike a typical vision transformer, the VSS module avoids positional embedding and chooses a streamlined structure without MLP phases, achieving a denser stack of blocks within the same depth budget.

### 2.3. Hybrid Attention Enhancement Mechanism (HAEM)

**Figure 3:**
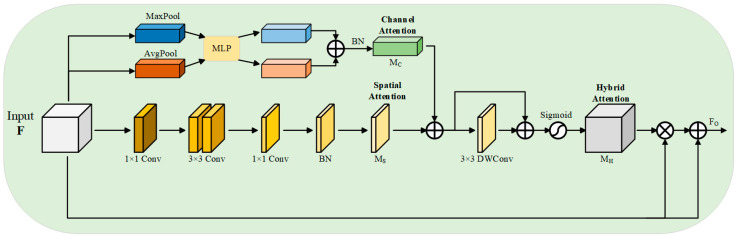
Hybrid attention enhancement mechanism HAEM.

The bottleneck of the network receives the output feature maps from the encoder, and it is difficult to capture the target accurately due to the many noise artifacts in breast ultrasound images, so this paper proposes a new module, HAEM, which receives the output features from the encoder, and through the organic combination of spatial attention [29] as well as channel attention [30], it captures and filters the more critical regions and features, which enhances the network’s ability to capture the target region and improves the accuracy of segmentation. HAEM contains two branches, channel attention and spatial attention, and the specific structure is shown in Figure 3.

Given the input feature $F\in {\mathbb{R}}_{\;⠀}^{C\times H\times W}$, a hybrid attention feature map $M_{H}^{\;⠀}\in {\mathbb{R}}_{\;⠀}^{C\times H\times W}$ is computed by HAEM, which is then weighted with the input features to obtain the final output feature map $F_{O}^{\;⠀}\in {\mathbb{R}}_{\;⠀}^{C\times H\times W}$, which is computed as:

(8) $$F_{O}^{\;⠀}=F+M_{H}^{\;⠀}⊗F$$

where ⊗ denotes the element-by-element multiplication between matrices.

The hybrid attention feature map $M_{H}^{\;⠀}$ is formed by aggregating channel attention $M_{C}^{\;⠀}\in {\mathbb{R}}_{\;⠀}^{C\times 1\times 1}$ and spatial attention $M_{S}^{\;⠀}\in {\mathbb{R}}_{\;⠀}^{1\times H\times W}$. The specific process can be expressed as follows:

(9) $$M_{H}^{\;⠀}=\sigma \left({\mathrm{DWConv}}_{\;⠀}^{3\times 3}\left(M_{C}^{\;⠀}+M_{S}^{\;⠀}\right)+\left(M_{C}^{\;⠀}+M_{S}^{\;⠀}\right)\right)$$

where σ is the Sigmoid activation function, and DWConv is a 3×3 depthwise convolution, which aims to enhance local relationships and cluster information.

In channel attention branching, each channel of the input features contains a specific feature response, and the relationship of each channel in the channel branching is fully utilized to aggregate the features in the channel. Firstly, global maximum pooling and global average pooling are performed on the input feature $F\in {\mathbb{R}}_{\;⠀}^{C\times H\times W}$ in spatial dimension, respectively, and two feature maps with a dimension of 1×1×C are obtained. Then the results of global maximum pooling and global average pooling are fed into a shared multilayer perceptron (MLP) for learning, respectively, to obtain two output feature maps of dimension 1×1×C. To reduce computational parameters, a dimensionality reduction operation is used in the MLP, the number of neurons in the first layer of the MLP is C/r, the activation function is ReLU, and the number of neurons in the second layer is C. Finally, the two outputs of the MLP are summed up, and then subjected to batch normalization (BN) to obtain the final channel attention map $M_{C}^{\;⠀}$, the specific formula of the above process is:

(10) $$M_{C}^{\;⠀}=\mathrm{BN}\left(\mathrm{MLP}\left(\mathrm{AvgPool}\left(F\right)+\mathrm{MaxPool}\left(F\right)\right)\right)$$

(11) $$\mathrm{MLP}\left(\mathrm{AvgPool}\left(F\right)\right)=W_{1}^{\;⠀}\left(W_{0}^{\;⠀}\left(F_{\mathrm{avg}}^{\;⠀}\right)+b_{0}^{\;⠀}\right)+b_{1}^{\;⠀}$$

(12) $$\mathrm{MLP}\left(\mathrm{MaxPool}\left(F\right)\right)=W_{1}^{\;⠀}\left(W_{0}^{\;⠀}\left(F_{\max}^{\;⠀}\right)+b_{0}^{\;⠀}\right)+b_{1}^{\;⠀}$$

where AvgPool denotes global average pooling, MaxPool denotes global maximum pooling, $F_{\mathrm{avg}}^{\;⠀}$ denotes the output feature of global average pooling, and $F_{\max}^{\;⠀}$ denotes the output feature of global maximum pooling, $W_{0}^{\;⠀}\in {\mathbb{R}}_{\;⠀}^{C/r\times C}$, $b_{0}^{\;⠀}\in {\mathbb{R}}_{\;⠀}^{C/r}$, $W_{1}^{\;⠀}\in {\mathbb{R}}_{\;⠀}^{C\times C/r}$, $b_{1}^{\;⠀}\in {\mathbb{R}}_{\;⠀}^{C}$.

In the spatial attention branch, this paper employs Dilated Convolution (DC) [31] to reduce the computational effort while expanding its sensory field to construct a more efficient spatial feature map than standard convolution. Specifically, the input feature $F\in {\mathbb{R}}_{\;⠀}^{C\times H\times W}$ is first projected to a ${\mathbb{R}}_{\;⠀}^{C/r\times H\times W}$ reduced dimension space using 1 × 1 standard convolution to facilitate the compression of the feature map in the channel dimension. Then the compressed feature map is convolved with two 3 × 3 dilated convolutions to effectively extract the context information. Finally, the feature map is again transformed into a feature map in ${\mathbb{R}}_{\;⠀}^{1\times H\times W}$ dimension using 1 × 1 standard convolution, and a batch normalization operation is performed on it to obtain the final output spatial attention feature map $M_{S}^{\;⠀}$, which is computed as:

(13) $$M_{S}^{\;⠀}=\mathrm{BN}\left({\mathrm{Conv}}_{3}^{1\times 1}\left({\mathrm{Conv}}_{2}^{3\times 3}\left({\mathrm{Conv}}_{1}^{3\times 3}\left({\mathrm{Conv}}_{0}^{1\times 1}\left(F\right)\right)\right)\right)\right)$$

After obtaining the required attention feature maps by channel attention branching and spatial attention branching, respectively, the spatial attention feature map and the channel attention feature map are summed up element by element, and then a deep convolution is performed to enhance the local relations, gather the more effective information, and construct the residual connections [32], and then activated by the Sigmoid function to obtain the hybrid attention features. Then, the hybrid attention features are element-wise multiplied with the input features to obtain an enhancement matrix, which is then added to the input features to obtain the output features of the HAEM at the bottleneck, i.e., the input features of the decoder.

### 2.4. Cross-Fusion Module (CFM)

**Figure 4:**
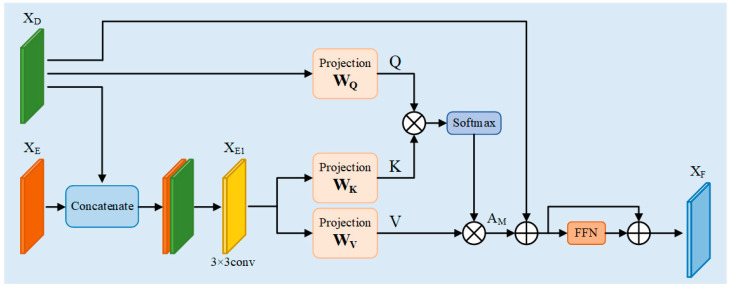
Cross fusion module CFM.

Skip Connection is particularly important in medical segmentation, which not only facilitates information fusion but also improves the efficiency of gradient propagation while preserving the underlying information. Usually, the encoder partially reduces the resolution gradually by downsampling to extract features, but the downsampling operation will lead to the loss of spatial detail information of the target, which will affect the segmentation performance. Therefore, jump-joining is necessary in image segmentation. Since the boundary of the breast ultrasound image is blurred and the target region is difficult to be precisely localized, the simple jump connection cannot cope with this more complex environment. Therefore, in this paper, a new module, CFM is designed, which takes the shallow features output from the encoder and the deep features output from the decoder as inputs, and effectively fuses the spatial detail information at the bottom layer with the semantic information at the top layer, which can further improve the accuracy and robustness of the segmentation results of the model. The specific structure of the CFM is shown in Figure 4.

The CFM first receives the same dimensional outputs $X_{E}^{\;⠀}\in {\mathbb{R}}_{\;⠀}^{C\times H\times W}$, $X_{D}^{\;⠀}\in {\mathbb{R}}_{\;⠀}^{C\times H\times W}$ from the encoder and decoder, respectively. Then, the two are spliced in the channel direction to obtain a feature map containing spatial and semantic information with dimension ${\mathbb{R}}_{\;⠀}^{2C\times H\times W}$. The number of channels is reduced to the original dimension by 3 × 3 convolution, and then the new feature map, i.e., $X_{E1}^{\;⠀}\in \in {\mathbb{R}}_{\;⠀}^{C\times H\times W}$, is obtained by the activation function ReLU as well as by normalization. The specific process is as follows:

(14) $$X_{E1}^{\;⠀}=\mathrm{ReLU}\left(\mathrm{BN}\left({\mathrm{Conv}}_{\;⠀}^{3\times 3}\left(\mathrm{concat}\left(X_{E}^{\;⠀},X_{D}^{\;⠀}\right)\right)\right)\right)$$

After obtaining the new feature map $X_{E1}^{\;⠀}$ that aggregates spatial and semantic information, it is fed into the linear projection layer for learning, along with the output feature $X_{D}^{\;⠀}$ of the decoder. $X_{D}^{\;⠀}$ is mapped to Q through the linear layer, and $X_{E1}^{\;⠀}$ is mapped to K and V. Then Q, K, and V are used as inputs for the dot-product attention computation, which proceeds as follows:

(15) $$\mathrm{Attention}\left(x\right)=\mathrm{softmax}\left(\frac{X_{D}^{\;⠀}W_{Q}^{\;⠀}{\left(X_{E1}^{\;⠀}W_{K}^{\;⠀}\right)}_{\;⠀}^{T}}{\sqrt{d_{k}^{\;⠀}}}\right)X_{E1}^{\;⠀}W_{V}^{\;⠀}$$

where $W_{Q}^{\;⠀}$, $W_{K}^{\;⠀}$ and $W_{V}^{\;⠀}$ are the weight matrices of the linear layers, softmax is the activation function, and $d_{k}^{\;⠀}$ is a constant that serves to prevent the result of the dot product from being too large.

In order to capture richer contextual information, increase the generalization ability of the network, and improve its sparsity, the multi-head operation [16] is introduced into the dot product operation and its dimensions are transformed, keeping the input and output dimensions unchanged, which can be expressed as follows:

(16) $$A_{M}^{\;⠀}=\mathrm{concat}\left({\mathrm{Attention}}_{1}^{\;⠀}\left(x\right),\ldots ,{\mathrm{Attention}}_{h}^{\;⠀}\left(x\right)\right)W_{O}^{\;⠀}$$

where concat is the concatenate operation and $W_{O}^{\;⠀}$ is the weight matrix, which is intended to keep the input and output constant throughout the attention computation process.

After obtaining the attention matrix $A_{M}^{\;⠀}$, residual concatenation is performed with the decoder output $X_{D}^{\;⠀}$ to obtain a new output R. This is then passed through the feed-forward network FFN [16] and the residual concatenation is used again to finally obtain the final output of the cross-fusion module $X_{F}^{\;⠀}$. The specific process can be expressed as follows:

(17) $$R=A_{M}^{\;⠀}+X_{D}^{\;⠀}$$

(18) $$X_{F}^{\;⠀}=R+\mathrm{FFN}\left(R\right)$$

where $X_{F}^{\;⠀}\in {\mathbb{R}}_{\;⠀}^{C\times H\times W}$, which is consistent with the input feature dimensions. FFN is a feed-forward neural network, i.e., it is a linear layer, and the input and output dimensions are kept constant, which can be expressed as follows:

(19) $$\mathrm{FFN}\left(x\right)=\max\left(0,xW_{1}^{\;⠀}+b_{1}^{\;⠀}\right)W_{2}^{\;⠀}+b_{2}^{\;⠀}$$

In the model of this paper, the output dimensions of each level of the encoder are $\frac{H}{4}\times \frac{W}{4}\times C$, $\frac{H}{8}\times \frac{W}{8}\times 2C$, $\frac{H}{16}\times \frac{W}{16}\times 4C$, and $\frac{H}{32}\times \frac{W}{32}\times 8C$, respectively, and the dimensions of each level of the decoder are $\frac{H}{16}\times \frac{W}{16}\times 4C$, $\frac{H}{8}\times \frac{W}{8}\times 2C$, and $\frac{H}{4}\times \frac{W}{4}\times C$, respectively. Each CFM receives the encoder outputs and decoder outputs with matching dimensions, respectively, and calculates the new feature $X_{F}^{\;⠀}$ through CFM, and then uses it as an input into the decoder module of the next level, and so on to achieve the final outputs, and then goes through the linear projection layer to achieve the final segmentation result map.

### 2.5. Loss Function

In breast ultrasound image segmentation, foreground as well as background need to be predicted, which is a pixel-by-pixel classification problem; the most common loss function is the cross-entropy loss function; in this paper, we use a weighted binary cross-entropy loss function, which can be expressed as:

(20) $$L_{\mathrm{wce}}^{\;⠀}=-\frac{1}{N}\sum_{i=1}^{N}\sum_{c=1}^{C}w_{c}*g_{i,c}\ln\left(p_{i,c}\right)$$

The cross-entropy loss function calculates the loss of each pixel equally, when the number of pixels in the foreground is much smaller than that of the background pixels, the loss of the background will be dominant, which leads to the model focusing too much on the background and ignoring the foreground; therefore, this paper combines the cross-entropy loss function as well as Dice loss function, which will effectively overcome the phenomenon of category imbalance, and also improve the spatial consistency. Dice loss function specifically can be expressed as:

(21) $$L_{\mathrm{wdice}}^{\;⠀}=1-\sum_{c=1}^{C}\frac{2w_{c}\sum_{i}^{N}p_{i,c}g_{i,c}}{\sum_{i}^{N}p_{i,c}^{2}+\sum_{i}^{N}g_{i,c}^{2}}$$

where N is the total number of pixels, C is the number of categories, $p_{i,c}^{\;⠀}$ denotes the predicted value, $g_{i,c}^{\;⠀}$ denotes the true value of the mask, and $w_{c}^{\;⠀}$ is the value of the weights assigned.

The final total loss is obtained by weighing the cross-entropy loss as well as the Dice loss, which is:

(22) $$L=0.4*L_{\mathrm{wce}}+0.6*L_{\mathrm{wdice}}$$

The Dice loss enhances the model’s inference for contiguous regions, while the cross-entropy loss ensures per-pixel classification accuracy. The combination of the two can improve the spatial consistency between the predicted regions while ensuring the classification accuracy at the pixel level.

## 3. Experiments

### 3.1. Material Preparation

#### 3.1.1. Environment Configuration

The experiments in this paper were conducted on Windows 11 operating system with Inter(R) Core (TM) i9-9900 CPU (Intel Corporation, Chandler, AZ, USA), NVIDIA GeForce RTX 2070 SUPER graphics card, and 8GB of GPU memory. The platform for model compilation was PyCharm 2023.3.4, and the framework used for model training was Pytorch 2.5.0 and CUDA 12.4 for acceleration. During the training process, the input size of the image is uniformly set to 224 × 224, and some hyperparameters of the model are set: the initial learning rate is 0.01, the momentum is 0.9, the training batch size is 8, the number of iterations is 300, and the optimizer is SGD.

**Figure 5:**
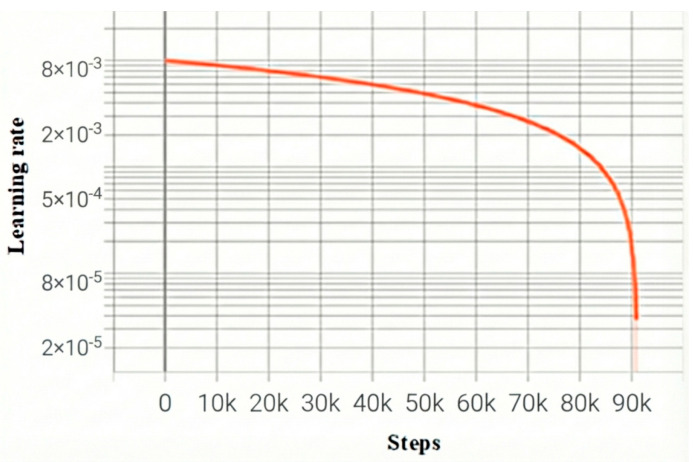
Model learning rate decay curve.

**Figure 6:**
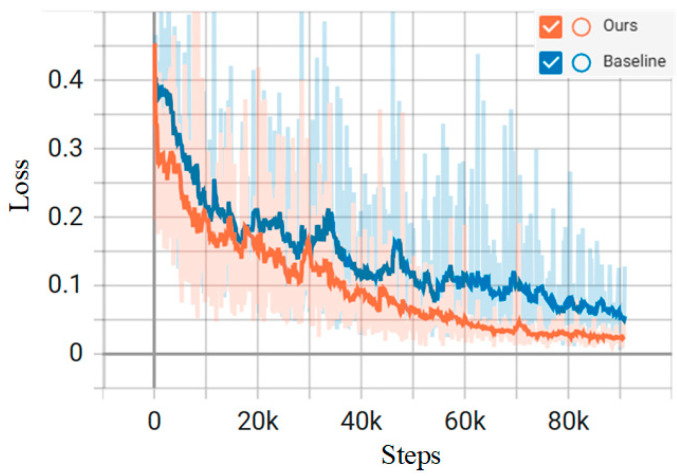
Model training loss function variation curve.

The learning rate decay curve, as well as the loss function change curve, during the training process are shown in Figure 5 and Figure 6, respectively, and the total loss, as well as the learning rate, is recorded once for each batch trained. The orange curve represents the proposed model, while the blue curve is the baseline model of Mamba. The horizontal coordinate step in Figure 5 and Figure 6 is the number of batches; the vertical coordinates, respectively, represent the learning rate and loss value of each batch of the model. As the model iterates, the model starts to converge when Step is 75k, i.e., about 250 training rounds.

Table 1 shows the comparison of the parameter counts and FLOPs between our model and several baselines, where the optimal results are bolded. As shown in the table, our model achieves optimal performance in terms of parameter count and computational complexity, with 21.76 million parameters and 39.19 GFLOPS, respectively. Compared to CNN- and Transformer-based models, we have reduced the parameter count while improving segmentation accuracy. In terms of computational complexity, our model is slightly higher than HCTNet and MambaUnet. This may be attributed to the incorporation of attention modules, which introduce quadratic computational complexity, resulting in a marginally higher computational load. Overall, our algorithm balances parameter counts and computational complexity while improving segmentation accuracy, demonstrating the model’s robust segmentation prediction performance and practical applicability.

#### 3.1.2. Datasets

**Figure 7:**
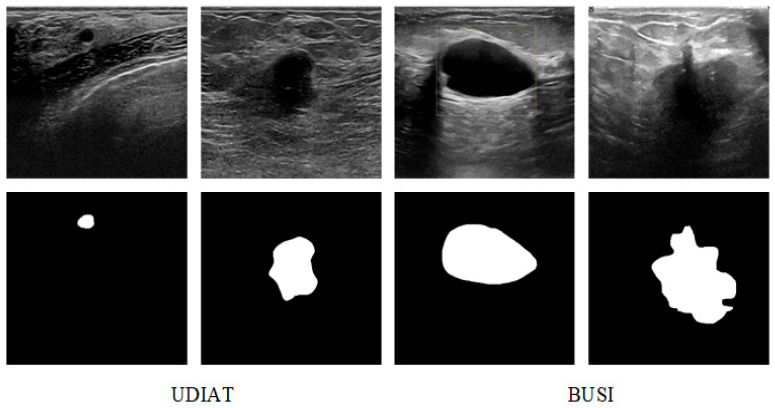
Examples of BUSI and UDIAT.

The datasets used in the proposed model are two breast ultrasound image segmentation datasets, BUSI [33] and UDIAT [13], as shown in Figure 7. The BUSI dataset contains 780 breast ultrasound images of female patients, which are classified into three categories: normal, benign tumors, and malignant tumors, and the datasets all contain segmentation mask maps for tumor cells. The UDIAT dataset contains 163 breast ultrasound images with detailed annotations, of which 53 are malignant tumors, and the remaining 110 are benign tumors.

**Figure 8:**
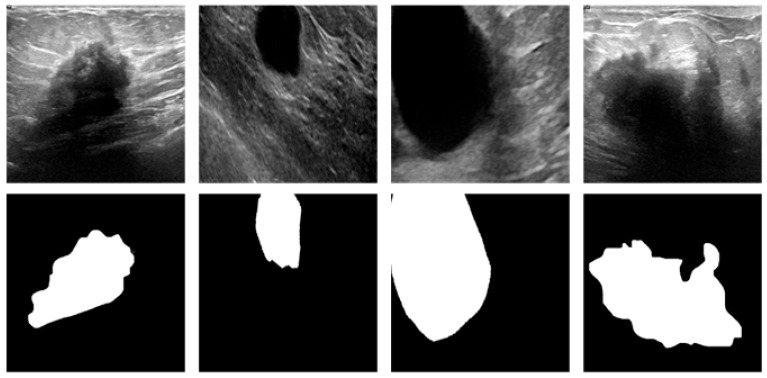
Examples of experimental datasets.

The mixed BUSI and UDIAT dataset is firstly divided into training and test sets in the ratio of 8:2. In order to expand the dataset samples to cope with more complex environments and to improve the robustness of the model, data enhancement was performed on the training set and test set using flipping, random cropping, and other methods, respectively, to obtain the new experimental dataset, as shown in Figure 8. The new experimental dataset contains 3028 ultrasound images as well as 3028 segmentation mask images. This not only solves the problem of insufficient training data but also simulates the complexity in many breast ultrasound images, improves the segmentation performance of the network, and improves the generalization ability of the model.

#### 3.1.3. Evaluation Metrics

To objectively evaluate the segmentation performance of the network model, we use the more common performance metrics in the field of medical image segmentation, which include the Dice Similarity Coefficient (Dice), the Hausdorff Distance (HD), the Precision (Pre), and the Recall (Rec).

Dice evaluates the similarity between two sets by calculating the ratio of their intersection and concatenation, which is an important performance evaluation index in segmentation tasks, and can objectively reflect the segmentation effect of the model on the target region; the higher the value, means that the segmentation result is more accurate. HD is a metric describing the degree of similarity between the two sets of points, which is used to measure segmentation accuracy at the boundary, and the lower the value, means that the segmentation effect is better. Pre refers to the proportion of correctly predicted positive samples among all predicted positive samples, and the higher the value, the higher the segmentation accuracy; Rec refers to the ratio of correctly predicted positive samples to the total number of real positive samples, and the higher the value, the better the segmentation effect. The formulas for the above indicators are, respectively:

(23) $$\mathrm{Dice}\left(x\right)=\frac{2\times TP}{2\times TP+FN+FP}$$

(24) $${\mathrm{HD}}_{95}\left(x\right)=\max{\left(\underset{a\in A}{\max}\;\underset{b\in B}{\min}\;d\left(a,b\right),\underset{b\in B}{\max}\;\underset{a\in A}{\min}\;d\left(a,b\right)\right)}_{95\%}$$

(25) $$\mathrm{Pre}\left(x\right)=\frac{TP}{TP+FP}$$

(26) $$\mathrm{Rec}\left(x\right)=\frac{TP}{TP+FN}$$

TP (True Positive) means that the model determines the same results as the true label, which are all positive samples. TN (True Negative) means that the model determines the same results as the true label, which are all negative samples. FP (False Positive) indicates that the model’s judgment result is a positive sample, while the true label is a negative sample. FN (False Negative) indicates that the model’s judgment result is a negative sample, while the true label is a positive sample. For the Hausdorff distance, the maximum distance is generally not selected. Instead, the distances are sorted from smallest to largest, and the top 5% are extracted as the target. The purpose is also to exclude some unreasonable distances and maintain the stability of the data. Therefore, the Hausdorff distance is also abbreviated as HD_95_.

### 3.2. Experimental Results

#### 3.2.1. Module Control Experiment

To investigate the rationality and effectiveness of the HAEM designed in this paper, controlled experiments are conducted with other attention modules on the experimental dataset.

The HAEM proposed in this paper is an effective aggregation of channel attention and spatial attention mechanisms, and local feature enhancement by deep convolution. Controlled experiments are conducted between HAEM and the spatial attention module (SAM), CAM, and the bottleneck attention module (BAM) [34] under the same base model, and the experimental results are shown in Table 2, where the optimal results are bolded.

As can be seen from Table 2, when only SAM or CAM is inserted at the bottleneck of the base model, the segmentation performance of the model performs poorly, with the lowest Dice coefficients and high HD_95_ indexes, which also indicates that it is totally insufficient to pay attention to and capture the features in only one dimension. The BAM combines spatial attention and channel attention to pay attention to the features in multiple dimensions at the same time, which has a better improvement, and the Dice coefficient and HD_95_ metrics reach sub-optimal. HAEM combines channel attention and spatial attention more effectively, and enhances the local relationship of features through deep convolution and residual connection, so that the segmentation performance of the model reaches its best, and both the Dice coefficient and the HD95 index reach their optimal, which are 73.89% and 23.21 mm, respectively. Thus, it can be shown that the HAEM proposed in this paper is reasonable and more effective than other attention mechanisms.

#### 3.2.2. Comparison of Different Algorithms

In order to verify the effectiveness of the proposed algorithm on breast ultrasound image segmentation, under the premise of using the same dataset, training equipment, and training strategy, this algorithm is compared with the mainstream medical segmentation algorithms of the present time, including the classical CNN-based medical image segmentation networks, Unet [8], FPN [10], and DeepLabv3+ [11], Transformer-based segmentation models, Transunet [17], Swin-unet [19], and HCTNet [35], and the Mamba-based segmentation models VMamba [22], MambaUNet [23].

Data augmentation is applied to datasets to simulate complex real-world scenarios such as image blurring and occlusion, thereby enhancing model robustness. Prior to this, we conducted comparative experiments using selected models on the original datasets (BUSI-only and UDIAT-only).

Table 3 and Table 4 present the comparison results of selected models on the BUSI and UDIAT datasets, respectively, with the optimal metrics highlighted in bold. As shown in the table, our model demonstrates outstanding performance across all metrics, with the Dice coefficient achieving optimal results. Due to the small sample size and inherent randomness of the original dataset, coupled with its clear images and minimal environmental influences, we applied data augmentation to expand the sample size while simulating complex environments. We then conducted experiments on the model using this new experimental dataset.

Table 5 gives the evaluation results of the proposed method with eight other segmentation methods on the experimental dataset consisting of BUSI and UDIAT, where the optimal metrics are bolded.

In Table 5, our proposed model is optimal in Dice, HD_95_, and Rec metrics, while the Pre reaches the second. Dice and Rec are higher than the suboptimal model by 0.85% and 1.40%, respectively, while the HD_95_ is lower than the suboptimal model by 1.76 mm. Specifically, the CNN-based segmentation models Unet and FPN perform poorly in terms of performance metrics, with poor segmentation results on the BUSI and UDIAT datasets. For Unet, its model complexity is low, and it is difficult to capture the detailed information of the image; it is also less capable of coping with complex environments. The FPN model adopts a feature pyramid structure, which may lose the feature information of the highest pyramid in the process of downsampling, and the FPN may reduce the multiscale expression ability when fusing features of different levels due to the semantic divide between different layers. The Deeplabv3+ has a great improvement in performance compared with Unet and FPN models, but due to its insufficient processing of boundary detail recovery and long-distance dependence of CNN, the performance of this model is not optimal.

The performance of the three major Transformer-based segmentation networks, Transunet, Swin-unet, and HCTNet, is significantly improved over the traditional CNN medical segmentation networks, and considerable progress has been achieved in the experimental dataset in various metrics, but there are still shortcomings. Combined with the advantages of CNN and Transformer, the Transunet model has more advantages in long-term modeling. However, Transunet does not further process image details, so it is difficult for this model to deal with complex environments with blurred boundaries. Swin-unet introduces a shift window mechanism based on the Transformer, which enhances the interaction between features and slightly improves various performance indicators. However, as this algorithm is a pure Transformer architecture, its computational quadratic complexity is high, and its ability to capture edge details is insufficient, resulting in its segmentation performance indicators not achieving the best. HCTNet, combined with a residual network and a Transformer, pays attention to the global and local characteristics of the model at the same time, which greatly improves the segmentation performance of the network, and various performance indicators rank among the top three. However, when dealing with a complex environment, the model can only roughly segment the lesion area, and there is still room for improvement in the boundary details.

The Mamba-based medical segmentation model considers the long-range dependent modeling capability while reducing the computational quadratic complexity, which significantly improves the accuracy of breast ultrasound image segmentation in all performance metrics. The VMamba model, as the underlying visual Mamba model, has a simpler structure; therefore, the segmentation capability is not outstanding. MambaUnet uses VMamba as the encoder and decoder and adopts a symmetric structure and skip connection, such as Unet; its performance indicators Dice and HD_95_ reached 75.19% and 22.04 mm, respectively, placing it in the second position. All the performance indexes of our proposed model have reached the top two, among which the most important performance indexes, Dice and HD_95_, are the best.

**Figure 9:**
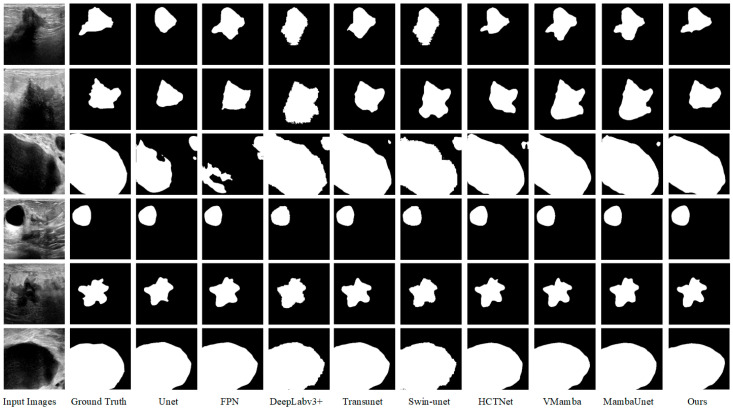
Visualization of breast ultrasound image segmentation results of different models.

Figure 9 shows the visualization of the segmentation results of eight different medical image segmentation models compared with the proposed model on the experimental dataset. Six images from the experimental dataset consisting of BUSI and UDIAT are selected, which include benign and malignant tumor images. The first column is the input breast ultrasound image, the second column is the real mask label, and the third column to the tenth column are the segmentation results of Unet, FPN, Deeplabv3+, Transunet, Swin-unet, HCTNet, VMamba, and MambaUnet models in turn. The last column shows the segmentation results of our proposed model.

Of course, as shown in the figure, our model also has some limitations. For particularly slender or curved lesions, segmentation may exhibit discontinuities or breaks. Although SSM theoretically excels at handling long sequences, inadequate capture of complex 2D geometric relationships in the scanning order or field-of-view design within 2D images may lead to insufficient modeling of continuous structures. In a small number of cases, boundary segmentation is inaccurate (either overly smooth or jagged), particularly when lesions of varying sizes coexist. Small lesions may be overlooked, or fine details of large lesion boundaries may be lost. In a small number of cases, boundary segmentation is inaccurate (either overly smooth or jagged), particularly when lesions of varying sizes coexist. Smaller lesions may be overlooked, while larger lesions may lose boundary details. Cross-attention may fail to optimally fuse the encoder (detail) and decoder (semantic) features. This could stem from Q/K/V projections failing to align key features, or the fusion process losing fine-grained spatial information. These findings clearly indicate that future work will focus on enhancing the model’s ability to represent complex structures and exploring more robust feature fusion strategies.

From Figure 9, it can be clearly seen that our proposed model is not only able to locate the lesion region more accurately, but also the model has a stronger ability to extract the edge detail features, and there are fewer cases of misclassification of the lesion region. Due to the lack of global context learning when processing local information, other segmentation models will be difficult to accurately find the lesion area in the segmentation results, and there is a high possibility of misjudgment in complex environments. Therefore, it can be considered that the segmentation algorithm proposed in this paper has more accurate segmentation results and stronger performance than other algorithms.

#### 3.2.3. Ablation Study

We propose HAEM and CFM, which can improve the segmentation performance of breast ultrasound images in complex environments such as blurred boundaries and more artifacts, by paying special attention to the critical regions and effectively fusing shallow and deep features. To further verify the validity of each module of the proposed segmentation algorithm, an ablation experiment was conducted on the experimental dataset. The control variable method was used in the ablation experiment, and the experiments were conducted in the case of the baseline network, adding only the hybrid attention enhancement mechanism, adding only the cross-fusion module, and the complete model, respectively. To investigate the impact of each layer of CFM on model performance, the CFM was inserted into the deepest layer (CFM-1) and the skip connections of each layer (CFM-3), respectively, for testing. The experimental results are shown in Table 6, in which the optimal results are bolded.

In Table 6, algorithm A represents the baseline network model composed of Mamba and CNN, algorithm B represents that only HAEM is introduced into the baseline model, algorithm C represents that CFM-1 is introduced into the baseline model, algorithm D represents that both HAEM and CFM-1 are introduced into the baseline model, and the complete model is that HAEM and CFM-3 are introduced into the baseline model. As can be seen in Table 6, Algorithm A performs the lowest in both Dice and Pre performance metrics, and HD_95_ also performs the highest. Compared with the basic network A, the Dice and Pre indicators of algorithm B are improved, while HD_95_ and Rec are slightly decreased. Compared with basic network A, network C is optimized in the other three performance indicators except for Rec. The Dice, Pre, and Rec performance indicators of network D are improved, and HD95 is reduced; the overall performance is suboptimal. The three performance indicators of the complete model, Dice, HD_95_, and Rec, are all optimal, and the Pre index is suboptimal. The segmentation effect of the complete model is the best, which is greatly improved compared with the baseline model.

**Figure 10:**
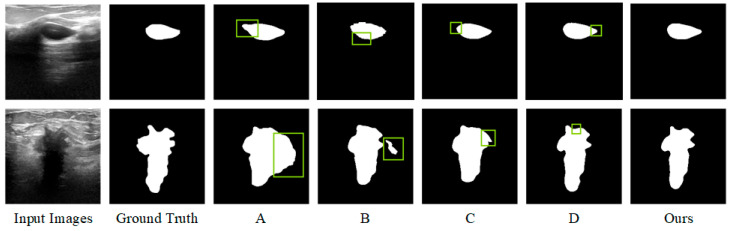
Visualization of ablation test results.

Figure 10 demonstrates a comparison of the visualization results of some ablation experiments on the dataset, where the green boxes mark the inaccurate regions. The baseline network A can only roughly localize the lesion region, and the misclassification range is large. While the lesion region localization is more accurate after adding the hybrid attention enhancement mechanism, the boundary contour of the segmentation target is not accurately grasped. After further adding the cross-fusion module, the model can not only accurately localize the lesion region but also control the details of the boundary more accurately, and the segmentation effect is optimized.

## 4. Conclusions

We propose a breast ultrasound image segmentation algorithm integrating Mamba-CNN and feature interaction, which effectively solves the problems caused by the complex environment, such as blurred boundaries and more artifacts in breast ultrasound images, and achieves better segmentation results; the Dice similarity coefficient and HD_95_ reach 76.04% and 20.28 mm, respectively. The proposed model effectively combines Mamba and CNN to refine local features while having excellent long-range modeling capabilities. We propose the HAEM, which enhances the attention to key regions of the features in the spatial and channel dimensions, filters out the effective information, excludes the redundant information, and effectively mitigates the artifact interference. Additionally, we design the CFM to effectively fuse the deep semantic information with the shallow spatial detail information through the interaction between the encoder and the decoder output features, to enhance the model’s grasp of the details of the boundary contour, and to effectively improve the segmentation accuracy of the model in the case of blurring of the image boundary. Comparison with other mainstream algorithms also validates the effectiveness of the proposed model. In future work, we will consider the lightweight research of the model to reduce the complexity of the model and improve the efficiency of segmentation while ensuring its segmentation accuracy. 

## Figures and Tables

**Table 1:** Model complexity comparison.

Method	Parameters/M	FLOPs/G
Unet	34.53	65.53
Transunet	92.23	148.06
Swin-unet	27.15	47.32
HCTNet	22.20	38.91
MambaUnet	27.84	**32.51**
Ours	**21.76**	39.19

**Table 2:** Comparison of results from different attention modules.

Base Model	Dataset	Module	Dice/% ↑	HD_95_/mm ↓	Pre/%	Rec/%
Mamba	Experimental dataset	CAM	72.98	25.43	75.11	76.06
		SAM	73.07	26.69	74.90	76.53
		BAM	73.37	24.44	**78.49**	75.81
		HAEM	**73.89**	**23.21**	78.32	**76.74**

**Table 3:** Comparison results of various models on BUSI.

Method	Dice/% ↑	HD_95_/mm ↓	Pre/%	Rec/%
Unet	75.23	13.47	82.19	77.91
Transunet	80.60	12.62	83.44	79.08
Swin-unet	80.80	11.93	**85.51**	83.90
HCTNet	81.24	10.53	83.59	82.12
MambaUnet	81.57	10.62	84.85	**83.94**
Ours	**82.89**	**10.38**	83.97	83.82

**Table 4:** Comparison Results of Various Models on UDIAT.

Method	Dice/% ↑	HD_95_/mm ↓	Pre/%	Rec/%
Unet	72.10	13.37	76.58	74.02
Transunet	74.61	12.63	80.28	76.63
Swin-unet	75.64	11.37	79.22	78.75
HCTNet	76.94	**9.97**	80.29	76.90
MambaUnet	77.95	10.57	80.85	**79.75**
Ours	**79.60**	9.99	**82.46**	79.46

**Table 5:** Comparison of results from different segmentation algorithms.

Algorithm	Dice/% ↑	HD_95_/mm ↓	Pre/%	Rec/%
Unet	70.72	28.52	77.00	71.48
FPN	71.26	26.15	78.51	72.58
DeepLabv3+	73.88	25.29	76.81	76.61
Transunet	73.83	23.71	79.95	73.60
Swin-unet	74.88	22.38	78.25	76.53
HCTNet	75.01	22.21	81.16	74.30
VMamba	73.02	23.77	80.13	72.44
MambaUnet	75.19	22.04	**81.22**	75.47
Ours	**76.04**	**20.28**	81.05	**76.87**

**Table 6:** Ablation test results.

Algorithm	HAEM	CFM-1	CFM-3	Dice/% ↑	HD_95_/mm ↓	Pre/%	Rec/%
A				74.36	22.81	77.53	75.74
B	√			75.00	21.19	80.75	74.91
C		√	√	75.52	21.58	81.04	74.69
D	√	√		75.99	21.06	**81.67**	76.06
Ours	√	√	√	**76.04**	**20.28**	81.05	**76.87**

## Data Availability

The original dataset BUSI can be accessed via the following link: https://doi.org/10.1016/j.dib.2019.104863.
